# Editorial on the Special Issue Entitled “Towards a Sustainable and Recyclable Future with Wood and Wood-Based Composites”

**DOI:** 10.3390/ma18214848

**Published:** 2025-10-23

**Authors:** Klementina Pušnik Črešnar, Olivija Plohl

**Affiliations:** 1 Faculty of Chemistry and Chemical Engineering, University of Maribor, Smetanova ul. 17, SI-2000 Maribor, Slovenia; 2 Faculty of Mechanical Engineering, University of Maribor, Smetanova ul. 17, SI-2000 Maribor, Slovenia

The constant search for more sustainable building materials is one of the key drivers of innovation in materials science and environmental technology. Wood and wood-based materials have long been researched, developed and used because they can compete with non-renewable polymers, metals and other materials in terms of their properties and performance, while having a more ecological and sustainable profile. The main advantages of wood and wood-based materials are their renewable nature, which results in them being biodegradable and carbon neutral, as well as their wide availability as one of the most abundant renewable resources on earth. However, their wider use as high-performance or industrial materials has historically been limited by some drawbacks, such as natural variability, susceptibility to biodegradation and fire, low thermal stability and limited recycling options, as well as problems associated with the reinforcement and bonding of wood. In recent years, progress has been made in overcoming these obstacles through the development of new or improved technologies that allow further development of wood and wood-based composites, both from a scientific point of view and in terms of actual performance and application potential.

While this research is a positive step towards the use of wood and wood-based composites as high-performance, sustainable and renewable alternatives to traditional building materials, many gaps remain. A crucial aspect for the adoption of these advanced materials, especially for commercial or industrial applications, is their scalability and economic feasibility. Many of the modification methods or manufacturing processes for composites developed in the laboratory have not yet been demonstrated on a large scale and at a competitive price. Another gap in this area is the lack of standardization for testing and performance evaluation. While this is an important step for benchmarking and comparison, it is also relevant for compliance with national and international regulations and guidelines. There is also a need to improve the understanding of the long-term behavior of these composites, particularly under combined or extreme conditions of moisture, UV exposure and biotic attack, to enable the development of reliable predictive models and design guidelines. In this context, it is important to note that although the concept of circular economy is increasingly discussed and applied in the context of wood and wood-based composites, its actual implementation is still lacking. Few studies have assessed the recyclability, biodegradability or life cycle impact of these advanced systems or attempted to integrate these factors into the development process. A potential problem with the use of wood and wood-based composites is the compatibility of natural fillers and biopolymers with existing industrial manufacturing and processing technologies. Factors such as surface roughness, moisture content or the presence of extractives can affect the quality of the end product and economic feasibility.

To fill these gaps, this Special Issue has compiled several original research articles that we believe make useful contributions in this direction. Many of the studies included in this Special Issue deal with the topic of wood modification. They show how chemical and/or mechanical treatments can be used to improve the durability of wood, which has long been seen as one of the major weaknesses of wood compared to other building materials. This is a particularly important finding as it represents a potential substitute for pressure-treated wood. Other articles look at the application of hybrid strengthening strategies, innovative composite lay-up techniques or unique fiber types that can be used to improve structural performance, providing data of interest to the construction and transportation industries. The topic of finger-jointing profiles and adhesive strategies in engineered wood is also of importance as it contributes to important improvements in terms of reducing stress concentrations and increasing the mechanical strength of joints and interfaces. The incorporation of recycled polymers and functionalized fibers or nanofillers, such as nanocellulose, is also included in some of the articles in this collection. This can be seen as evidence of how these types of approaches are able to achieve sustainability and performance at the same time, especially in the areas of packaging. Moisture-induced damage and acoustic monitoring are also among the degradation mechanisms studied, contributing to the understanding of the impact of environmental conditions on the performance of materials and the development of more suitable materials. Finally, the use of CO_2_ laser processing for cutting wood or wood-based composites is also analyzed and the importance of controlling roughness and surface irregularities in the production process is discussed.

In summary, the research results presented in this Special Issue provide a comprehensive overview of the current innovations in the field of wood and wood-based material technologies and provide both a critical assessment of the current state of knowledge and promising new insights for future research. It should be emphasized that further progress requires the development of fully bio-based composite systems, i.e., systems based solely on renewable resources without compromising material strength, thermal stability or durability. Consideration of life cycle analysis and environmental modeling should be a priority in the development of new materials to avoid replacing one environmental problem with another. Design for disassembly, reuse and recycling should also be a guiding principle, in line with the emerging concept of a circular economy. The development of smart wood-based materials capable of self-healing, sensing or thermoregulation could be another potential area for future exploration, opening up new applications in architecture, transportation or smart packaging. Widespread adoption will require the introduction of global standards and certifications that define performance expectations, safety parameters and environmental limits. Finally, digital manufacturing technologies such as machine learning, process automation or digital twin models have the potential to significantly improve production systems and quality control in the wood-based materials industry.

